# Prevalence of Internet Addiction Disorder and Its Correlates Among Clinically Stable Adolescents With Psychiatric Disorders in China During the COVID-19 Outbreak

**DOI:** 10.3389/fpsyt.2021.686177

**Published:** 2021-09-16

**Authors:** Zong-Lei Li, Rui Liu, Fan He, Shu-Ying Li, Yan-Jie Zhao, Wu-Yang Zhang, Yao Zhang, Teris Cheung, Todd Jackson, Yi-Lang Tang, Yu-Tao Xiang

**Affiliations:** ^1^Department of Psychiatry, Xiamen Xianyue Hospital, Xiamen, China; ^2^Beijing Key Laboratory of Mental Disorders Beijing Anding Hospital, The Advanced Innovation Center for Human Brain Protection, The National Clinical Research Center for Mental Disorders, School of Mental Health, Capital Medical University, Beijing, China; ^3^Department of Psychiatry, The First Affiliated Hospital of Zhengzhou University, Zhengzhou, China; ^4^Unit of Psychiatry, Department of Public Health and Medicinal Administration, & Institute of Translational Medicine, Faculty of Health Sciences, University of Macau, Macao, Macao SAR, China; ^5^Centre for Cognitive and Brain Sciences, University of Macau, Macao, Macao SAR, China; ^6^Institute of Advanced Studies in Humanities and Social Sciences, University of Macau, Macao, Macao SAR, China; ^7^School of Nursing, Hong Kong Polytechnic University, Hong Kong, Hong Kong SAR, China; ^8^Department of Psychology, University of Macau, Macao, Macao SAR, China; ^9^Department of Psychiatry and Behavioral Sciences, Emory University, Atlanta, GA, United States; ^10^Mental Health Service Line, Atlanta VA Medical Center, Decatur, GA, United States

**Keywords:** adolescent patients, COVID-19, internet addiction, psychiatric disorder, Chinese

## Abstract

**Background:** Since the Coronavirus disease 2019 (COVID-19) pandemic emerged, Internet usage has increased among adolescents. Due to this trend, the prevalence of Internet addiction disorder (IAD) may have increased within this group. This study examined the prevalence of IAD and its correlates among clinically stable adolescents with psychiatric disorders in China during the COVID-19 outbreak.

**Method:** A multi-center, cross-sectional study was carried out between April 29 and June 9, 2020 in three major tertiary mental health centers in China. IAD and depressive symptoms were assessed using the Internet Addiction Test (IAT) and the 9-item Patient Health Questionnaire (PHQ-9), respectively.

**Results:** A total of 1,454 adolescent psychiatric patients were included in final analyses. The prevalence of IAD was 31.2% (95% CI: 28.8–33.6%) during the COVID-19 pandemic. A multiple logistic regression analysis revealed that poor relationships with parents (*P* < 0.001, OR = 2.34, 95%CI: 1.49–3.68) and elevated total PHQ-9 scores (*P* < 0.001, OR = 1.19, 95%CI: 1.16–1.21) were significantly associated with higher risk for IAD while longer daily physical exercise durations (*P* = 0.04, OR = 0.67, 95%CI: 0.46–0.98) and rural residence (*P* = 0.003, OR = 0.62, 95%CI: 0.46–0.85) were significant correlates of lower risk for IAD.

**Conclusions:** IAD was common among adolescent patients with clinically stable psychiatric disorders during the COVID-19 pandemic; regular physical exercise, healthy relationships with parents and fewer symptoms of depression were associated with lower risk within this population.

## Introduction

The Coronavirus disease 2019 (COVID-19) pandemic is an international public health emergency that has negatively impacted many aspects of human life worldwide (1, 2). Because COVID-19 is highly contagious, a range of emergency public health measures has been adopted such as universal masking, social distancing, lockdowns, school closures, and public transportation suspension, all of which might have effects on the mental health and behavior of affected people, including adolescents (3–8). For instance, large-scale social quarantine/isolation during the pandemic with “forced” overexposure to the Internet may increase the risk for Internet addiction disorder (IAD) (9); additionally, adolescents typically spent more time on the Internet to study, play games, and chat with friends during the pandemic (10); these experiences have received growing attention as influences on IAD in adolescents during the COVID-19 pandemic.

IAD is among the most common mental health problems in adolescents (11). A previous study reported that the prevalence of IAD was 19.8% among mainland Chinese adolescents based on the Young's Internet Addiction Test (IAT) (12). A meta-analysis found the pooled prevalence of IAD was 11.3% in Chinese university students (13). A recent study on IAD during the COVID-19 epidemic found the prevalence of IAD was 2.68% based on the IAT while the corresponding figure for problematic internet use was 33.37% in a sample of 2,050 school-age children and adolescents in China (10). In another study on IAD among 1,060 junior high school students in Taiwan during the COVID-19 outbreak, the prevalence of IAD was 24.4% based on the Chen Internet Addiction Scale (14).

Regarding correlates of IAD, psychiatric disturbances including major depressive disorder (MDD), bipolar disorder (BD), insomnia, and attention-deficit hyperactivity disorder (ADHD) are common comorbid diagnoses (15–18). Prior studies have reported the prevalence of IAD that among child and adolescent psychiatric patients ranged from 11.3 to 24.1% (17, 19, 20). To date, however, little is known about rates of IAD in clinically stable adolescents with psychiatric disorders during the COVID-19 pandemic.

The Internet has been used widely among patients with psychiatric disorder *via* computers, smartphones or other devices (21–24). The internet offers non-traditional options for access to information and communication, and has been linked to reduced stress, anxiety and/or depressive symptoms in select studies (21, 25, 26). For many clinically stable adolescent patients with psychiatric disorders, maintenance pharmacotherapy is required. On the one hand, during the COVID-19 pandemic, these adolescents have limited access to mental health services, and the internet has been used as a helpful alternative resource (27) that could prolong time on the internet. On the other hand, overuse of the internet without effective control measures could result in IAD and contribute to exacerbations in disturbances among adolescents with psychiatric disorders (28). Previous studies have found that excessive internet use in the form of an addiction is related to the perpetuation of social anxiety, exacerbations in emotional distress and increased interference with daily functioning (29).

In order to reduce potential negative consequences of IAD for daily life and academic performance of psychiatrically vulnerable adolescents living through a pandemic, it is important to understand its frequency and correlates. Therefore, we conducted this study to examine the prevalence of IAD and its associated factors in clinically stable mainland Chinese adolescents with psychiatric disorders during the COVID-19 outbreak.

## Methods

### Patients and Study Sites

A multi-center, cross-sectional study was carried out between April 29 and June 9, 2020 in three major tertiary mental health centers for children and adolescents, located in the northern (Beijing), southern (Fujian province), and central regions of China (Hunan province, Hubei province). These hospitals represent a range of clinical settings in China. Due to the risk of transmission, traditional face-to-face interviews could not be conducted. Instead, following other studies (12, 27) data were collected using the WeChat-based Questionnaire Star application (Changsha Renxing Science and Technology, Shanghai, China). WeChat is a widely used social communication app with more than 1 billion users in China. To be eligible, participants were (1) aged between 10 and 17 years (the age range of adolescents used in participating hospitals), (2) outpatients receiving maintenance treatment for psychiatric disorders, and (3) “clinically stable” based on (i) judgments of treating psychiatrists and (ii) changes in psychotropic medication dosages of <50% during the past 3 months following previous operationalizations in the literature (30, 31), and (4) enrolled after providing personal verbal assent accompanied by guardians' written informed consent. Participants' principal psychiatric disorder according to International Statistical Classification of Diseases and Related Health Problems, 10th Revision (ICD-10; (32) were recorded in an electronic medical record system and confirmed by their treating psychiatrists. This study was approved by research ethics committees of the respective hospitals.

### Measurements

A pre-designed data form was used to collect information about basic socio-demographic background and clinical characteristics, including age, gender, household (urban/rural), status as only child, guardians' personal income, major medical conditions, principal psychiatric diagnosis (i.e., major depressive disorder (MDD)/bipolar disorder (BD)/ attention deficit hyperactivity disorder (ADHD)/ other diagnoses), perceived academic pressure, relationship with parents, concern about COVID-19, daily physical exercise, mass media use, difficulty seeing psychiatrists, treatment adherence and illness relapse during the COVID-19 outbreak. Treatment adherence was evaluated by a standardized question (“Degree to which the adolescent insisted on taking medicine during the COVID-19 outbreak?”), with options of “poor,” “fair,” and “good.” Illness relapse was assessed by a standardized question (“Degree to which the adolescent has had a relapse during the COVID-19 outbreak?”), with options of “No,” “Symptom worsening, but no relapse”, and “relapse.” Responses to questions on treatment adherence and illness relapse were confirmed by treating psychiatrists. Based on the results of a pilot study, those with assessment time s of less than 2 min were removed as a measure of quality control.

Internet addiction was assessed using the Chinese version of the Internet Addiction Test (IAT) (33, 34). The IAT comprises 20 items, each of which includes response options ranging from 1 (“rarely”) to 5 (“always”). This scale has been validated and widely used to screen internet addiction in Chinese adolescents (34, 35). IAT total scores of ≥50 are considered to reflect “IAD” (35, 36). Severity of depressive symptoms was assessed using the Chinese version of 9-item Patient Health Questionnaire (PHQ-9) (37, 38). Total PHQ-9 scores range from 0 to 27, with higher scores indicating more severe depressive symptoms (39–41). The PHQ-9 has satisfactory psychometric properties among adolescents (39, 41). In the current sample Cronbach's alpha value of PHQ-9 was 0.93.

### Data Analysis

Data were analyzed using SPSS software, version 24.0. Comparisons of social-demographic and clinical characteristics between patients with vs. patients without IAD were performed using chi-square tests, independent samples *t*-tests, or Mann-Whitney *U* tests, as appropriate. A multiple logistic regression analysis with the “enter” method was conducted to explore independent correlates of IAD status. All measures with significant differences in univariate analyses were entered as independent variables, and IAD was the dependent variable. The significance level was set as 0.05 (two-tailed).

## Results

A total of 1,570 adolescent patients were invited; of these, 1,454 met all inclusion criteria; their data were subjected to analyses. On the basis of setting, 381 patients (26.2%) were recruited from in Beijing Anding Hospital (Beijing), 576 (39.6%) from the First Affiliated Hospital of Zhengzhou University (Henan province), 243 (16.7%) from Xiamen Xianyue Hospital (Fujian province), and 254 (17.5%) from other parts of China. The prevalence of IAD was 31.2% (95% CI: 28.8–33.6%) in the current sample. Socio-demographic information and clinical characteristics of respondents are presented in Table 1.

**Table 1:** Socio-demographic and clinical characteristics of adolescent patients.

**Variable**	**Total**		**Non IAD**		**IAD**				
	**(** ***N*** **=** **1,454)**		**(** ***N*** **=** **1,000)**		**(** ***N*** **=** **454)**		**Univariate analyses**		
	***N***	**%**	***N***	**%**	***N***	**%**	**χ^**2**^**	**df**	***P***
Gender (male)	564	38.8	434	43.4	130	28.6	28.67	1	**<0.001**
Rural residence	642	44.2	462	46.2	180	39.6	5.44	1	**0.02**
Only child status	584	40.2	403	40.3	181	39.9	0.02	1	0.88
Major medical conditions	74	5.1	53	5.3	21	4.6	0.29	1	0.59
Primary psychiatric diagnosis							30.56	3	**<0.001**
MDD	759	52.2	479	47.9	280	61.7			
BD	123	8.5	85	8.5	38	8.4			
ADHD	70	4.8	61	6.1	9	2.0			
Others	502	34.5	375	37.5	127	28			
Perceived academic pressure							26.08	2	**<0.001**
Low	212	14.6	159	15.9	53	11.7			
Fair	717	49.3	523	52.3	194	42.7			
High	525	36.1	318	31.8	207	45.6			
Relationship with parents							77.70	2	**<0.001**
Good	537	36.9	435	43.5	102	22.5			
Fair	695	47.8	454	45.4	241	53.1			
Poor	222	15.3	111	11.1	111	24.4			
Concern with COVID-19							22.17	2	**<0.001**
Very concerned	360	24.8	276	27.6	84	18.5			
Moderately concerned	800	55.0	549	54.9	251	55.3			
No or minimal concern	294	20.2	175	17.5	119	26.2			
Daily physical exercise							22.76	2	**<0.001**
<30 min/day	1,062	73.0	693	69.3	369	81.3			
30–60 min/day	297	20.4	233	23.3	64	14.1			
More than 60 min/day	95	6.5	74	7.4	21	4.6			
Mass media use for COVID-19							2.642	2	0.267
No or very few	477	32.8	315	31.5	162	35.7			
Sometimes	660	45.4	460	46	200	44.1			
Often	317	21.8	225	22.5	92	20.3			
Difficulty seeing psychiatrists during COVID-19 pandemic							9.40	2	**0.009**
No or very few	1,146	78.8	797	79.7	349	76.9			
Sometimes	264	18.2	182	18.2	82	18.1			
Often	44	3.0	21	2.1	23	5.1			
Treatment adherence during COVID-19 pandemic							39.35	2	**<0.001**
Poor	529	36.4	330	33.0	199	43.8			
Fair	187	12.9	108	10.8	79	17.4			
Good treatment adherence	738	50.8	562	56.2	176	38.8			
Illness relapse in COVID-19							85.65	2	**<0.001**
No	656	45.1	520	52.0	136	30.0			
Symptom worsening, but no relapse	519	35.7	343	34.3	176	38.8			
Relapse	279	19.2	137	13.7	142	31.3			
Guardians' personal income (RMB3000 and above/mon)	942	64.8	654	65.4	288	63.4	0.53	1	0.47
	**Mean**	**SD**	**Mean**	**SD**	**Mean**	**SD**	***t/Z***	**df**	***P***
Age (years)	14.73	1.94	14.76	2.00	14.65	1.78	1.01	1,452	0.32
PHQ-9 Total	8.51	8.52	5.19	6.88	15.83	7.12	−22.12	–^a^	**<0.001**
IAT Total	41.21	19.17	30.13	8.97	65.61	11.56	−63.64	1,452	**<0.001**

a *Mann-Whitney U test; Bolded values: <0.05; COVID-19, coronavirus disease 2019; MDD, major depressive disorder; BD, bipolar disorder; ADHD, attention deficit hyperactivity disorder; PHQ-9, the 9-item patient health questionnaire; IAT, internet addiction disorder; SD, standard deviation*.

Univariate analyses indicated gender, residence, principal psychiatric diagnosis, PHQ-9 total scores, perceived academic pressure, relationship with parents, concern of COVID-19, daily physical exercise, difficulty seeing psychiatrists, treatment adherence and illness relapse during the COVID-19 outbreak significantly differed between adolescent patients with IAD and those without IAD (all *P*-values < 0.05).

A multiple logistic regression analysis revealed that poor relationship with parents (*P* < 0.001, OR = 2.34, 95%CI: 1.49–3.68) and higher PHQ-9 total scores (*P* < 0.001, OR = 1.19, 95%CI: 1.16–1.21) were significant correlates of higher risk for IAD while longer daily physical exercise durations (P = 0.04, OR = 0.67, 95%CI: 0.46–0.98) and rural residence (*P* = 0.003, OR = 0.62, 95%CI: 0.46–0.85) were associated with significantly lower risk of IAD (Table 2).

**Table 2:** Independent correlates of IAD by multiple logistic regression analysis.

**Variables**	**Multiple logistic regression analysis**			
	***P***	**OR**	**95% CI**	
			**Lower**	**Upper**
Male gender	0.14	0.79	0.58	1.08
Rural residence	**0.003**	0.62	0.46	0.85
**Principal psychiatric diagnosis**				
MDD	–	1	–	–
BD	0.14	1.47	0.88	2.47
ADHD	0.28	0.63	0.28	1.45
Others	0.61	0.92	0.66	1.28
**Perceived academic pressure**				
Low	–	1	–	–
Fair	0.32	0.80	0.52	1.24
High	0.17	0.73	0.46	1.15
**Relationship with parents**				
Good	–	1	–	–
Fair	0.06	1.38	0.98	1.94
Poor	** <0.001**	2.34	1.49	3.68
**Concern with COVID-19**				
Very concerned	–	1	–	–
Moderately concerned	0.24	1.24	0.87	1.78
No or minimal concerned	0.22	1.31	0.85	2.02
**Daily physical exercise**				
<30 min/day	–	1	–	–
30–60 min/day	**0.04**	0.67	0.46	0.98
More than 60 min/day	0.07	0.56	0.29	1.06
**Difficulty seeing psychiatrists during COVID-19 pandemic**				
No or very few	–	1	–	–
Sometimes	0.71	0.93	0.64	1.36
Often	0.31	1.56	0.67	3.64
**Treatment adherence during COVID-19 pandemic**				
Poor	–	1	–	–
Fair	0.12	1.44	0.91	2.29
Good treatment adherence	0.96	1.01	0.71	1.43
**Illness relapse in COVID-19**				
No	–	1	–	–
Symptom worsening, but no relapse	0.83	1.04	0.74	1.45
Relapse	0.51	0.87	0.57	1.33
**PHQ-9 Total**	**<0.001**	1.19	1.16	1.21

*Bolded values: <0.05; CI, confidential interval; OR, odds ratio; COVID-19, coronavirus disease 2019; MDD, major depressive disorder; BD, bipolar disorder; ADHD, attention deficit hyperactivity disorder; PHQ-9, the 9-item patient health questionnaire; Residence was controlled for study sites*.

## Discussion

To the best of our knowledge, this is the first study to examine the prevalence and correlates of IAD among clinically stable adolescents with psychiatric disorders in China during the COVID-19 outbreak. We found the prevalence of IAD in this sample was 31.2% (95% CI: 33.6–28.8%). This rate is higher than figures from previous studies using the IAT. For example, the prevalence of IAD was 10.4% among 1,059 Chinese adolescents in Anhui province (42), 26.5% for 6,468 adolescents in Guangzhou (43), and 23.7% (95% CI: 22.1–25.2%) among 2,892 adolescents in a multicenter survey of China (12). Additionally, the prevalence of IAD in this study was higher than rates reported for adolescents with psychiatric disorders. For example, the prevalence of IAD was 20.7% using the Compulsive Internet Use Scale in 111 adolescents receiving inpatient psychiatric care in Austria (17), 24.1% using the IAT among psychiatric adolescent patients in Turkey (20), 11.3% using a standardized instrument for internet addiction in adolescent psychiatric inpatients from Germany (19), and 12.9% (95% CI: 7.6–19.7%) using the IAT in 132 adolescent outpatients with autism spectrum disorder and/or attention-deficit hyperactivity disorder in Japan (44).

Apart from the influence of differences in study sites, sample sizes, measurement tools and/or timeframes of performed research, there are several potential reasons for the relatively high prevalence of IAD in our sample. First, similar to substance use disorders (45), the risk to develop IAD is associated with amount of exposure. Compared to pre-pandemic eras, adolescents, particularly those with psychiatric disorders, may have had more exposure to the internet in active (e.g., playing games, and communications with friends) or passive (e.g., doing homework, and taking online classes) forms during the COVID-19 pandemic. In line with this contention, recent studies have found school-age children spent significantly longer time on the Internet during the pandemic (10, 46). Second, particular residual psychiatric symptoms including lack of confidence, poor self-esteem, and lack of social skills in daily life may contribute to an increased likelihood of excessive Internet use or the development of IAD (35, 47). Some studies have found that certain psychiatric disorders in adolescents (e.g., ADHD, MDD, anxiety disorders) correlate with increased risk of IAD (15, 16, 48, 49). Previous studies on coping have also suggested that the Internet is a common way to release emotions and escape from or avoid real-life problems; such strategies may lead to excessive internet usage, resulting in IAD among adolescents with psychiatric disorders (29, 35, 50).

Similar to previous findings (13, 51, 52), we found that the adolescents in rural areas had a lower risk of IAD. Compared with adolescents living in rural areas, adolescents living in urban areas have greater ease in accessing the Internet through smartphones and computers (13, 53). Furthermore, adolescents living in rural areas more often engaged in housework or farming activities in order to relieve family burdens (54); these experiences can reduce available free time, potentially contributing to less computer exposure (13, 53).

In our multivariate analysis, having 30–60 min/day of physical exercise emerged as a significant correlate of lower risk for IAD among adolescent psychiatric patients during the COVID-19 pandemic. This finding dovetails with a number of studies that underscore regular physical activity and exercise as protective factors against excessive internet use, stress, and negative emotions within general adolescent samples (11, 55–57). Coupled with these results, our findings provide foundations for the hypothesis that regular daily physical exercise helps adolescent psychiatric patients to reduce stress, anxiety and fear of the COVID-19 pandemic, and functions as a protective factor against IAD.

Logistic regression analysis results also indicated adolescent psychiatric patients who had poor relationships with their parents were at significantly higher risk for IAD. This finding converges with other evidence linking IAD with family conflict, an overprotective parenting style, reduced parental supervision, and less family communication (58–60). Conversely, more harmonious parent-adolescent relationships have positive associations with emotion regulation capacities that serve as a developmental resource that lessens problem behaviors of children (61). In the context of the COVID-19 outbreak, poor parent-adolescent relationships may not have buffering effects against loneliness and insecure feelings of adolescent psychiatric patients (3) who are cut-off from direct personal contact with others and more prone to excessive Internet use and IAD.

Finally, the multivariate model identified more severe depressive symptoms as a correlate of significantly higher risk for IAD among adolescents with psychiatric disorders. This finding is also consistent with other studies (21, 35, 62). Due to fear, helplessness, and stress resulting from the COVID-19 pandemic and a range of preventive measures (e.g., mass lockdowns, home quarantine, prolonged school closure and public transportation suspension) (3) that foster physical isolation between people, adolescents who have more depressive symptoms may use the Internet to obtain social support, enhance self-esteem, and/or distract themselves from negative affect or other stressors in their lives, potentially increasing their risk for IAD. Notably, however, previous studies have documented bidirectional relationships between depression and IAD. On one hand, IAD alone is a risk factor for certain psychiatric disorders (63, 64). On the other hand, the occurrence of psychiatric disorders could increase the likelihood of IAD (65).

Several limitations of this study should be noted. First, as alluded to above, the cross-sectional study design precludes the capacity to make causal statements about the status of IAD as a cause or consequence of associated experiences. Second, for ethical reasons, this study included clinically stable patients, limiting the generalizability of findings to currently less stable patients, a group for whom appropriate clinical care is a priority relative to completing a research study. Third, due to lack of widely accepted diagnostic criteria for IAD, various tools have been used in different studies. Consequently, direct comparisons of IAD patterns between studies and conclusions about prevalence in this literature must be made with caution. Fourth, severity of psychiatric disorders (e.g., BD, ADHD, and others) which may be associated with Internet use behaviors among adolescents and certain potentially relevant correlates of IAD (e.g., residual psychiatric symptoms, social support, sleep quality, physical health, social distancing, duration of lockdowns, time spent on internet for study purposes), were not measured in light of the heavier response burdens that would have been incurred upon unpaid research participants.

In conclusion, this study indicated IAD is common among clinically stable adolescent psychiatric patients during the COVID-19 pandemic. However, within the sample, regular daily physical activity and better perceived relationships with parents emerged as significant behavioral correlates of reduced risk for IAD while elevations in depressive symptoms were correlated with significantly increased risk for IAT. Considering the negative consequences of IAD on daily life and academic performance of adolescents with psychiatric disorders, preventive measures, routine screening, and timely treatments should be undertaken for those at risk during the COVID-19 pandemic. Furthermore, findings are potentially useful for health authorities and mental health professionals toward developing guidelines, designing preventive programs and implementing policies that help clinically stable adolescents with psychiatric disorders to prevent IAD. For example, prevalence data provide useful foundations for policies that may aid in curbing excessive internet use among adolescent psychiatric patients. As well, apart from medication treatment for residual psychiatric symptoms, certain psychosocial interventions such as cognitive behavior therapy and family counseling to improve adolescent-parent relationships could be offered to adolescents at risk for IAD in rehabilitation programs (66). Finally, mental health professionals should be funded to develop appropriate online services to help clinically stable patients reduce feelings of shame, stress and loneliness related to IAD and regulate their internet usage (6).

## Data Availability Statement

The Research Ethics Committees of participating hospitals that approved the study prohibits the authors from making the research dataset of clinical studies publicly available. Readers and all interested researchers may contact Dr. Yu-Tao Xiang (Email address: xyutly@gmail.com) for details. Dr. Xiang will apply to the Research Ethics Committee of participating hospitals for the release of the data.

## Ethics Statement

The studies involving human participants were reviewed and approved by Beijing Anding Hospital, Xiamen Xianyue Hospital, and First Affiliated Hospital of Zhengzhou University. Participants provided personal verbal assent accompanied by guardians' written informed consent.

## Author Contributions

Study design: Z-LL and Y-TX. Data collection, analysis, and interpretation: Z-LL, FH, S-YL, Y-JZ, W-YZ, YZ, and TC. Drafting of the manuscript: RL, Y-TX, and Y-LT. Critical revision of the manuscript: TJ. All authors approved the final version for publication.

## Funding

This study was supported by the Beijing Municipal Science & Technology Commission (Z181100001718124), the Beijing Talents Foundation (Grant No.: 2017000021469G222), the University of Macau (MYRG2019-00066-FHS), the Suzhou Key Medical Center for Psychiatric Diseases (Szzx201509), and the Health and Family Planning Key Talents Training Project of Fujian Province (2017-ZQN-93).

## Conflict of Interest

The authors declare that the research was conducted in the absence of any commercial or financial relationships that could be construed as a potential conflict of interest.

## Publisher's Note

All claims expressed in this article are solely those of the authors and do not necessarily represent those of their affiliated organizations, or those of the publisher, the editors and the reviewers. Any product that may be evaluated in this article, or claim that may be made by its manufacturer, is not guaranteed or endorsed by the publisher.
